# Microbiome analysis in a pediatric cohort of inflammatory bowel disease supports the rational of fecal microbiome therapy

**DOI:** 10.1186/2194-7791-1-S1-A23

**Published:** 2014-09-11

**Authors:** Aysefa Doganci, Rebecca L Knoll, Claudius U Meyer, Ulrike Kullmer, Fred Zepp, Stephan Gehring

**Affiliations:** Zentrum für Kinder und Jugendmedizin, Pädiatrische Immunologie, Universitätsmedizin Mainz, Mainz, Germany; Pädiatrische Gastroenterologie, Universitätsmedizin Mainz, Mainz, Germany

## Aims

In recent years, the role of gut flora in inflammatory bowel diseases (IBD), including ulcerative colitis (UC) and Crohn´s Disease (CD) has become focus of intense research. The working hypothesis is that an altered microbiota causes mucosal inflammation in a genetically susceptible individual. Understanding the microbiota´s role in the pathogenesis of the disease is essential for new IBD treatments aimed in shifting the intestinal bacterial flora back to a physiological homeostasis, particularly relevant for children not responding to conventional therapy.

## Methods

24 children, 6 with UC, 6 CD and 12 siblings without disease pattern of IBD were included in this study. Children were between 11-18 years and had no antibiotic treatment for the last 3 month before fecal sample collection. Frozen stool samples were delivered to EMBL, Heidelberg, where further proceedings carried out, including DNA extraction and next generation sequencing (NGS) of the samples.

## Results

The phylogenetic composition of 24 pediatric samples were investigated and compared with the human MetaHit project. The obtained data corroborated previous findings from adult samples. In detail, our IBD cohort displayed a lower bacterial diversity than the control group without disease pattern of IBD. In addition, we observed, that the enterotype distribution in children with IBD were missing the enterotype 2.

## Conclusion

We observed that children with IBD have changes in the bacterial composition of feces with less bacterial diversity. These observations support the rational of transplanting the fecal microbiota from a healthy donor into an individual with IBD. The latter might restore the healthy gut microbiota in the patient’s diseased colon, leading to the resolution of symptoms, thus acting as a novel therapeutic approach for children with IBD.

